# Prevalence of problem alcohol use among patients attending primary care for methadone treatment

**DOI:** 10.1186/1471-2296-10-42

**Published:** 2009-06-11

**Authors:** Niamh Ryder, Walter Cullen, Joseph Barry, Gerard Bury, Eamon Keenan, Bobby P Smyth

**Affiliations:** 1 UCD School of Medicine and Medical Science, Coombe Healthcare Centre, Dublin, Ireland; 2 Department of Public Health and Primary Care, Trinity College, Dublin, Ireland; 3 Department of Public Health, Health Services Executive, Dublin, Ireland; 4 Addiction Services, Health Services Executive, Dublin, Ireland

## Abstract

**Background:**

Problem alcohol use is associated with adverse health outcomes among current or former heroin users and primary care is providing methadone treatment for increasing numbers of this population. This study aimed todetermine the prevalence of problem alcohol use among current or former heroin users attending primary care for methadone treatment and to describe the socio-demographic characteristics and health service utilisation characteristics associated with problem alcohol uses.

**Methods:**

We conducted a cross sectional survey of patients sampled from a national database of patients attending general practice for methadone treatment. Participants were recruited by their general practitioner and data was collected using an interviewer-administered questionnaire, which included the Alcohol Use Disorders Identification Test ('AUDIT'), with a score of >7 considered abnormal (ie 'AUDIT positive cases') and socio-demographic, medical and substance use characteristics.

**Results:**

We interviewed 196 patients (71% of those invited, 31% of those sampled, 11% of the national database). The median age was 32 years, 55% were hepatitis C positive, 79% had used illicit drugs in the previous month and 68% were male. Sixty-eight 'AUDIT positive' cases were identified (prevalence of 35%, 95% CI = 28–41%) and these were more likely to have attended a local Emergency Department in the previous year (p < 0.05) and less likely to have attended a hospital clinic in the previous year (p < 0.05). Twenty-seven (14%) scored 20 or higher indicating possible alcohol dependence.

**Conclusion:**

Problem alcohol use has a high prevalence among current or former heroin users attending primary care for methadone treatment and interventions that address this issue should be explored as a priority. Interventions that address problem alcohol use in this population should be considered as a priority, although the complex medical and psychological needs of this population may make this challenging.

## Background

Problem alcohol use is associated with adverse health outcomes and is a common problem among people who use heroin and other illicit opiates, with one-third of opiate-using patients attending a specialist addiction clinic in the UK being identified as 'problem drinkers, hazardous drinkers or alcohol dependent' on screening [1].

The adverse health implications of problem alcohol use among patients on methadone treatment include:

Injecting drug users are at high risk of liver disease resulting from hepatitis C infection and in Ireland, 62–81% of injecting drug users are hepatitis C positive [2]. Problem alcohol use is an important factor in determining poor prognosis among people with hepatitis C infection as lifetime consumption of alcohol and occasional heavy alcohol drinking have been shown to play an additive role in determining progression to hepatic cirrhosis [3]. In addition, excessive alcohol intake is associated with increased HCV-RNA levels and elevated hepatic aminotransferase levels [4].

Problem alcohol use also affects addiction treatment, by impacting negatively on treatment outcomes [5] and by affecting methadone metabolism [6] and the dose of methadone that must be prescribed [7]. Alcohol is an important factor in fatal opiate overdose [8], especially if a person has ingested other central nervous system depressants [9].

In Ireland, addressing problem alcohol use has recently been identified as a priority for population health. Since 1995, there has been a dramatic increase in alcohol related harm in Ireland, with this harm observed across a range of health and social indicators [10]. While no published data has reported the prevalence of problem alcohol use among patients on methadone treatment, a recently published qualitative survey of patients attending primary care for methadone treatment has suggested problem alcohol use may be a common problem, with patients possibly even substituting opiate dependency with alcohol dependence [11].

The role of primary care in addressing problem alcohol use has been described. A review of brief, multi-contact behavioural counselling interventions among adult patients attending primary care found that such interventions reduced the average of drinks per week by 13–34% and increased the proportion drinking at moderate or safe levels by 10%–19% and concluded that such interventions were feasible and potentially highly effective components of an overall public health approach to reducing alcohol misuse [12].

As alcohol-related harm is such a key issue for population health and as current or former drug users are at particular risk of alcohol-related harm (and therefore benefit from therapeutic interventions) further data regarding problem alcohol use among this group are needed. With primary care increasingly involved in providing addiction care and with an increased evidence base regarding the role of primary care in addressing problem alcohol use, data regarding problem alcohol use among current or former heroin users attending primary care are needed. This paper is the first to report on a problem alcohol use among patients attending primary care for treatment.

The primary objective of this study therefore was to determine the prevalence of problem alcohol use among current or former heroin users attending primary care for methadone treatment. The secondary objective was to identify the socio-demographic characteristics and health service utilisation characteristics of those patients with problem alcohol use.

## Methods

### Setting

Heroin use has been a longstanding problem issue in Dublin city, where an estimated 16.0 per thousand of the adult population currently use illicit opiates [13]. In Ireland, although the number of new cases presenting for addiction treatment has increased in recent years (59.0 per hundred thousand of the adult population during 1998 to 69.1 per hundred thousand of the adult population during 2002), the number of these new cases reporting opiate addiction has fallen (855 in 1998 to 729 in 2002) [14].

In Ireland, addiction treatment is currently provided by specialist addiction treatment services, including a central Drug Treatment Centre Board, regional addiction centres, community-based projects (satellite clinics) and by primary care. The most recently published data at the time of this study indicated that 7845 patients were treated for problem drug use by these agencies in 2002, with opiates the most common drug for which people attended for treatment (86% of total) [14].

In recent years, the number of GPs who provide addiction treatment in the form of methadone prescribing has increased in Ireland, the UK and elsewhere in the EU [15-19]. To prescribe methadone in Ireland, GPs must complete specific training and are subject to clinical audit, with GPs who provide methadone treatment for 15 or more patients subject to more regular audit and advanced training ('level 2' GPs) [20]. This system is analogous to the 'GPs with a special interest' ('GPWsi') model currently operating in the UK [21].

Initiation of methadone treatment is only permitted in specialist addiction clinics or by 'level 2' GPs [20]. Opiate users with complex histories or significant comorbid difficulties are initially cared for in a specialist addiction clinic and their care transferred to general practice when stable.

### Subjects, power calculation and sampling

A national database of patients being prescribed methadone was used to identify potential subjects. Since the introduction of legislation regulating the prescribing of methadone in Ireland, it has not been possible to dispense methadone to a patient unless his or her name is entered on this database, which is held by the Drug Treatment Centre Board (Central Methadone Treatment List). Each patient's name is accompanied by the name of a corresponding prescribing doctor and dispensing pharmacy [20].

At the time of the study, the national database indicated 2585 patients were attending primary care for methadone treatment. We estimated that sampling 629 patients (25% of all those on the database) would yield a 95% confidence interval of +/- 4% around a prevalence estimate of patients who meet the criteria for 'problem drinkers, hazardous drinkers or alcohol dependent drinkers' of 33%. This allowed for a non-response rate of 30%, which we felt was a conservative estimate given the proposed study methodology. As cases were sampled individually from the national database, we did not control for the clustering effect in determining our sample size.

### Study instrument

An interviewer-administered questionnaire containing the primary study instrument, the Alcohol Use Disorders Identification Test ('AUDIT') was used. The 'AUDIT' is a 10-item screening questionnaire, whose applicability in detecting patients with alcohol problems in primary care has been validated. It contains questions relating to frequency of drinking, typical quantity, frequency of heavy drinking, impaired control over drinking, increased salience of drinking, morning drinking, guilt after drinking, blackouts, alcohol-related injuries and others concerned about drinking [22].

### Data collection

The interviews were conducted between February 2006 and October 2007. At the time of the study, addiction treatment services in Ireland were organised in four geographical areas and data collection was staggered to allow data be collected on a region by region basis.

A researcher with no input into clinical care administered the questionnaire by interview, an approach allowing clarification of ambiguous answers and administration of the questionnaire to patients with poor literacy skills [22]. Additional self-reported data on a range of variables which could be associated with problem alcohol use were also recorded, including: socio-demographic and substance use characteristics, addiction care and other medical issues.

### Data analysis

Data was analysed using Statistical Packages for the Social Sciences (SPSS) version 12.0. Each question on the 'AUDIT' questionnaire has a standard set of responses from which to chose, each scored from 0 to 4 and a total score of 8 or above was considered abnormal (ie 'AUDIT positive cases), with a score of 8–15 indicative of 'hazardous' alcohol use, 16–19 indicative of 'harmful' alcohol use and 20 or above indicative of possible alcohol dependence [22].

Analytical techniques included Pearson's chi squared test and Fisher's exact test statistic (used in the case of small sample sizes) to determine the significance of associations between categorical variables. Odds ratios and their 95% confidence intervals (CI) were used to describe the relationship between subject characteristics and abnormal scores on AUDIT questionnaire. Multivariate analyses were performed using logistic regression, with variables found to be significant on univariate testing entered into the regression equation.

### Ethical considerations

A step-wise approach to patient recruitment was used. Patients attending primary care for methadone treatment were randomly sampled from the Central Treatment List. The GP of each potential participant was then invited to participate in the study, asked to approach the named patient to determine if s/he was willing to meet with the researcher. Potential participants were provided with written information regarding the study and if willing to participate, an appointment to meet with a member of the research team.

At this meeting, patients were provided with further explanation on the study, the nature of the questions that would be asked and they were encouraged to express any concerns or issues requiring clarification. In particular, it was made explicit to patients that non-participation in the study would not compromise the care they receive. When all such issues had been explained to the patient's satisfaction, he or she was asked to consent to participate in the study by signing a consent form.

Participation in the study by GPs and patients was on a voluntary basis. No inducements to participate were offered and refusal to participate did not compromise patient care.

While information from individual interviews was not reported to the patient's GP, all patients were advised to discuss the issue of problem alcohol use and any issues that had been raised by the interview with their GP. It was also made explicit that the interview with the researcher was for research purposes and did not constitute any aspect of their clinical care and merely represents part of a research project. All data was anonymised and any details that could potentially identify individuals were removed.

The study was approved by the Research Ethics Committee of the Irish College of General Practitioners

## Results

### Sample recruitment and socio-demographic characteristics

**Figure 1:**
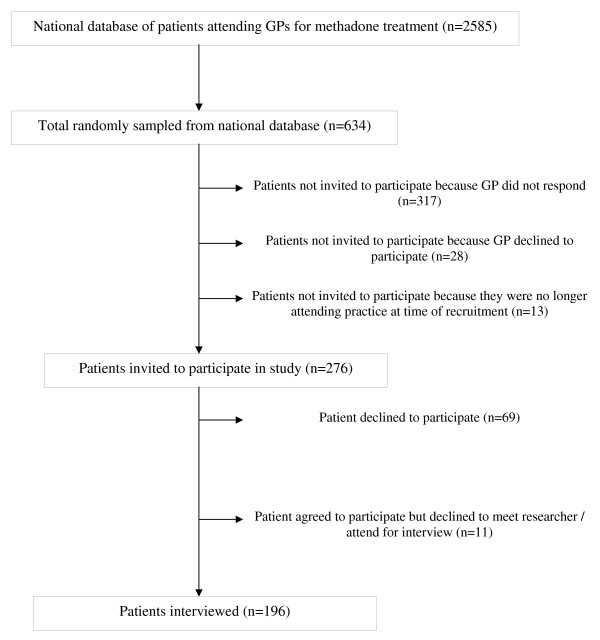
**Patient recruitment**.

We randomly sampled 634/2585 patients (25% random sample) from the national database, but 358 were not invited to participate in the study because their GP declined to participate in the study, did not respond to two written invitations and five or more follow up telephone contacts or because at the time of recruitment, they were no longer attending the practice for methadone treatment. Therefore, 276 patients were invited to participate in the study (10.7% of those on national database), of whom we interviewed 196 (71% participation rate among those invited but 31% of random sample) (see Figure 1 – patient recruitment).

Table 1 compares the characteristics of the sample recruited for this study with most recent data on patients attending general practice for methadone treatment and patients attending for treatment of problem drug use in Ireland [14,23,24]. The sample recruited for this study had a median age of 32 years and 133(68% were male). A total of 88(45%) indicated they were currently in full or part time employment, 116(59%) reported they were in a relationship and 77(39%) reported they were living with their partner/spouse at the time of interview. One hundred and forty (71%) reported they had children and 85 (43%) reported they were currently living with their children. Thirty seven (19%) reported they lived in their own home, 83(42%) lived in rented accommodation, 62(32%) lived in their parents'/other family member's home and three (2%) reported they were homeless and 11 did not answer.

**Table 1:** Characteristics of sample population compared to other samples of patients attending for methadone/addiction treatment in Ireland.

***Variable***		***This study***	***Patients attending GPs for methadone treatment (Barry, Personal Communication, 2005)***	***Patients attending general practice for methadone treatment ***[23,24]	***All patients attending for problem drug use treatment in Ireland ***[14]
Demographics	% male	68%	68%	72%	71%
	
	% employed	45%	NA	NA	25.8%
	
	% homeless	2%	NA	NA	3%
	
	Median age at time of study (range)	32(20–55)	32(18–62)	28*	25.8(17.2–40.7)
	
	Median age of first heroin use (range)	18(13–52)	NA	NA	NA
	
	Median age of first injecting drug use	19(13–39)	NA	19.4*	19(15–28)

Bloodborne virus infections	% (of those tested) who were known to be HCV positive	55	NA	73	NA
	
	% (of those tested) who were known to be HIV positive	5	NA	9	NA

Geographical location of practice by health region	East Coast Area	38(30%)	333(13%)	NA	NA
	
	Northern Area	39(18%)	751(29%)	NA	NA
	
	South West Area	115(45%)	1325(51%)	NA	NA
	
	Rest of Ireland	4(11%)	197(8%)	NA	NA

NA: Data not available/not reported.

* indicates mean value as median not reported.

### Problem alcohol and substance use

Of the 196 interviewed, 68 (35%) scored positive on the AUDIT screening instrument, indicating hazardous, harmful or dependent alcohol use. Of these 'AUDIT positive' cases, 33 scored 8–15 (indicative of 'hazardous' alcohol use), 8 scored 16–19 (indicative of 'harmful' alcohol use) and 27 scored 20 or higher (indicative of possible alcohol dependence).

All participants had a history of heroin use. The median age of first heroin use was 18 years. A total of 149 (76%) indicated they had ever injected drugs, with 19 years the median age of first injecting drug use. All were being prescribed methadone by their GP at the time of the interview (median dose 75 mgs daily), with 48 months the median length of time they had been attending that specific practice and 48 months also the median length of time they had been prescribed methadone on this treatment episode. When asked 'which term best described' their 'type of treatment', 36(18%) indicated they were on 'detoxification' with the remainder indicating they were on 'maintenance' treatment.

One hundred and fifty-five (79%) had used at least one other illicit substance in the previous month, with 119(61%) using cannabis, 49(25%) using heroin, 22(11%) using illicit methadone, 42(21%) using illicit benzodiazepines, 26(13%) using cocaine, 6(3%) using crack cocaine and 3(2%) using amphetamines. Nine (5%) participants reported injecting drug use, heroin in all cases, in the month before interview.

### Other medical issues

With regard to bloodborne virus infections, 188(96%) claimed to know their hepatitis C status, 114(58%) had a test in the previous 12 months and 107(55%) indicated they were antibody positive. A total of 187(95%) claimed to know their HIV status, 111(57%) had a test in the previous 12 months and 10(5%) indicated they were positive.

When asked to report contact with other health agencies in the year before the interview 39(20%) reported they had attended a hospital clinic, with hepatitis C (29 respondents), psychiatry (three respondents) and back pain (two respondents) being the clinics most commonly attended. Thirty-four respondents (17%) reported they had attended a local hospital Emergency Department during this time, with trauma and/or musculoskeletal injuries (14 respondents), liver problems (three respondents) and chest pain (two respondents) being the most commonly cited reasons.

### Factors associated with problem alcohol use

Table 2 presents data exploring potential associations between problem alcohol use and patient characteristics, including sociodemographic characteristics, problem substance use or other medical issues. On univariate analysis, AUDIT positive cases were significantly more likely to have attended a local Emergency Department in the last year, to have used illicit benzodiazepines in the last month or to have used illicit amphetamines in the last month. They were significantly less likely to consider themselves on maintenance (instead of detoxification) treatment with methadone and less likely to have attended a scheduled hospital clinic appointment in the last year.

**Table 2:** Factors associated with scoring greater than 7 on AUDIT questionnaire.

Factor	AUDIT positive cases (n = 68)	AUDIT negative cases (n = 128)	Odds ratio (95% CI)	Chi ^2 ^statistic (p value)
*Sociodemographic characteristics*				

Age under 33	38(56%)	71(56%)	1.02(0.56–1.84)	0.00(0.96)

Male gender	50(74%)	83(65%)	1.51(0.79–2.88)	1.54(0.22)

Attending GP for 48 months or less	36(53%)	55(52%)	1.06(0.59–1.91)	0.34(0.85)

Currently in full or part-time employment	29(46%)	59(48%)	0.91(0.50–1.68)	0.09(0.76)

Living in own home	11(18%)	26(21%)	0.78(0.36–1.71)	0.39(0.54)

Living with at least one relative	41(65%)	75(62%)	1.17(0.62–2.20)	0.82(0.66)

Living with a partner/spouse	27(66%)	50(67%)	0.96(0.43–2.16)	0.01(0.93)

Has children	39(62%)	90(74%)	0.58(0.30–1.11)	2.77(0.10)

Living with children	22(56%)	63(70%)	0.56(0.26–1.21)	2.24(0.14)

*Problem substance use*				

Current methadone treatment episode 48 months or less	38(56%)	67(52%)	1.15(0.64–2.08)	0.22(0.64)

Methadone dose of 60 mgs or less daily	25(37%)	35(27%)	1.55(0.83–2.89)	1.86(0.17)

Maintenance methadone treatment *	50(74%)	110(86%)	0.46(0.22–0.95)	4.56(0.03)

First used heroin at 18 years or younger	44(65%)	80(63%)	1.08(0.58–1.99)	0.06(0.81)

Have injected heroin ever	47(69%)	102(80%)	0.57(0.29–1.12)	2.72(0.10)

*Other medical issues*				

Tested for hepatitis C in the previous 12 months	41(60%)	73(57%)	1.14(0.63–2.08)	0.19(0.66)

Perceived to be hepatitis C positive	36(58%)	71(56%)	1.07(0.58–1.98)	0.05(0.82)

Tested for HIV in the previous 12 months	40(59%)	71(56%)	1.15(0.63–2.08)	0.20(0.65)

Perceived to be HIV positive	3(5%)	7(6%)	0.86(0.21–3.44)	0.05(0.83)

Attended a hospital clinic in the last year*	7(10%)	32(25%)	0.34(0.14–0.83)	6.03(0.01)

Attended a local Emergency Department in the last year*	17(25%)	17(13%)	2.18(1.03–4.61)	4.25(0.04)

*Use of illicit substances in the previous month*				

Any illicit substance	57(84%)	98(77%)	1.59(0.74–3.41)	1.42(0.23)

Cannabis	46(68%)	73(57%)	1.58(0.85–2.92)	2.10(0.15)

Heroin	21(31%)	28(22%)	1.60(0.82–3.10)	1.92(0.17)

Methadone	10(15%)	12(9%)	1.67(0.68–4.09)	1.27(0.26)

Benzodiazepines*	21(31%)	21(16%)	2.28(1.14–4.56)	5.53(0.02)

Cocaine	12(17.6%)	14(11%)	1.75(0.76–4.02)	1.74(0.19)

Crack cocaine	1(2%)	5(4%)	0.37(0.04–3.21)	0.89(0.35)

Amphetamines*	3 (4%)	0(0%)	N/A	5.74(0.02)

* association at < 0.05 significance level;

** association at < 0.01 significance level.

On multivariate analysis, AUDIT positive cases were still significantly more likely to have attended a local Emergency Department in the last year (Wald statistic = 5.8; p < 0.05) and less likely to have attended a hospital clinic in the last year (Wald statistic = 4.3; p < 0.05). Among the 107 who reported they were hepatitis C positive, problem alcohol users were significantly less likely to have attended a specialist hepatology clinics, odds ratio (95% CI) = 0.17 (0.05–0.58); chi^2 ^= 10.9 (p < 0.005).

## Discussion

### Summary of main findings

This study is the first to present data on the prevalence of problem alcohol use among opiate dependent patients attending primary care for methadone treatment. We observed a prevalence of 35%, which is equivalent to rates reported among opiate dependent patients treated in specialist settings. We also observed high rates of illicit substance use, hepatitis C infection and health service utilisation. Twenty-seven (14%) scored 20 or higher on the AUDIT questionnaire, which is indicative of possible alcohol dependence.

We found problem alcohol use to be associated with illicit drug use, attendance at a local Emergency Department and having not attended a specialist hospital clinic.

### Strengths and limitations of the study

A number of methodological issues must be considered in interpreting our findings. Our participation rate was lower than had been anticipated and Figure 1 outlines the reason for this. While ethical considerations determined the initial approach to potential participants should be made by their GP, this step had a major impact on recruitment. Therefore, the possibility of selection bias can not be discounted, with 345(54% of possible total) not even being approached to participate in the study because their GP either declined to participate in the study or did not respond to repeated contacts by the research team. In Ireland, this is the first time that potential participants were prospectively recruited on the basis of random sampling from a national database of methadone prescribing. Our experience with recruitment for this study would therefore support more targeted approaches to recruitment, with practices/GPs who wish to participate in similar research being identified before sampling, with sampling occurring at the level of the individual practices. While this would have implications for power calculations, with these needing to take account of the clustering effect, it would lead to a higher participation rate and a more efficient use of resources.

While we have no data on the GPs who did not participate in the study, we have compared patients who participated in the study with all patients attending general practice for methadone treatment at the time of the study and found our study sample to be comparable in terms of age and gender (Barry, Personal Communication, 2005, see Table 1). The sample was also comparable to most recent data on patients attending general practice for methadone treatment and patients attending for treatment of problem drug use in Ireland, in terms of: gender, age of first injecting, proportion that were homeless (see Table 1). However, the sample is older, has a higher proportion that report being employed and a lower proportion reporting they are HCV or HIV positive [14,23,24]. Table 1 also indicates that while our sample is representative in respect of two geographical regions, one region (East Coast area) is over-represented and one is under-represented (Northern Area) (Barry, Personal Communication, 2005).

Notwithstanding these similarities, our sample represents only 31% of those randomly sampled from the national database. This sample may represent the more 'stable' end of the spectrum, in which case the possibility for our findings to underestimate the prevalence of problem alcohol use can not be discounted.

As our data essentially reports point prevalence of problem alcohol use (using the AUDIT questionnaire) and its correlates, it offers no perspective on the natural history of problem alcohol use or its temporal relationship to problem drug use. In that respect, longitudinal studies which explore the relationship between problem drug use and problem alcohol use over time will help us understand factors associated with individuals developing this high-risk co-dependency. We also note the previously described limitations of using the AUDIT questionnaire alone to determine treatment options in predicting treatments outcomes [25].

### Comparison with existing literature

Screening and treating patients attending general practice for problem alcohol use is important for many reasons, including: the high prevalence of hepatitis C and risk of advanced liver disease [2,3], its negative impact on addiction treatment outcomes [5] and its role in fatal opiate overdose [8]. Our observed prevalence of problem alcohol was comparable to what has been reported in more high-risk populations, i.e. patients attending addiction clinics [1] and hospital in-patients [26].

This is a cause for concern as according to methadone prescribing regulations in Ireland, one might expect patients attending primary care for methadone treatment to have less severe addiction problems [27]. Work conducted elsewhere supports this view, indicating patients attending primary care for methadone treatment were more likely to be employed and to have less severe addiction problems than those attending specialist addiction clinics [28].

Our finding that problem alcohol use was not significantly associated with age or duration in treatment disagreed with our earlier exploratory work which suggested patients may substitute opiate dependence with alcohol dependence [11]. Instead, we found concurrent abuse of other substances was more common among problem alcohol users, with alcohol therefore forming part of a 'polysubstance misuse' pattern – a finding in keeping with work elsewhere [29]. This highlights the importance of problem alcohol use being addressed in conjunction with possible use of and/or addictions to other substances.

Other medical issues were common among our sample, with 55% reporting they were HCV positive, 5% reporting they were HIV positive, 20% reporting they had attended a hospital clinic and 17% reporting they had attended a local hospital Emergency Department in the previous year. This high prevalence of hepatitis C infection reinforces why addressing problem alcohol use among current or former drug users is so important.

The high contact rate with local hospital Emergency Departments supports recently published data which highlights problem alcohol use and problem substance use as important factors in Emergency Department utilisation [10,30]. Among our sample, this Emergency Department utilisation could be explained by their greater involvement in high risk behaviours.

We observed a high contact rate with out patient secondary care among our sample and this would indicate patients attending primary care for methadone treatment have complex medical needs that need to be addressed in addition to their primary addiction issues. We also found that people with problem alcohol use were less likely to engage with secondary care than people who did not report problem alcohol use. Specifically, they were less likely to have attended a specialist hospital clinic and, if hepatitis C positive, were less likely to have attended specialist hepatology clinics.

### Implications for future research and clinical practice

Many therapeutic interventions to address problem alcohol use have been described and evidence supports the role of screening, further assessment, brief interventions, more intensive treatments and alcohol-focussed specialist treatment [31,32] and the role of primary care based 'brief interventions' [33]. However, it is difficult to see whether this evidence would translate to the care of problem alcohol use among patients attending general practice.

Although screening and treating for problem alcohol has been identified as a potentially important element of methadone treatment programmes [34], especially for the most vulnerable clients [35], to date the implementation of no such intervention in primary care has been described. Our findings suggest that patients attending primary care for methadone treatment should be screened for problem alcohol use and should have access to care interventions that address coexisting problem alcohol use. With 14% of our sample reporting possible alcohol dependence, access to more specialised interventions, through integration with secondary care, would also appear important.

However, this study indicates that complex medical and psychological problems often coexist among this population and may pose additional challenges in delivering evidence based interventions that address problem alcohol use. In that regard, further research to determine existing care practices and to identify barriers to screening and treatment for problem alcohol use among patients on methadone treatment in primary care will be necessary to inform future service delivery.

## Conclusion

This study is the first to present data on the prevalence of problem alcohol use among opiate dependent patients attending primary care for methadone treatment and documents a high prevalence among this population. Interventions that address problem alcohol use in this population should therefore be considered as a priority, although the complex medical and psychological needs of this population may make implementing such interventions a challenge.

## Competing interests

The authors declare that they have no competing interests.

## Authors' contributions

WC conceived the study, WC, GB, JB, EK and BS authored the proposal, JB carried out the sampling, NR conducted the interviews, WC, NR and BS analysed the data. WC drafted the manuscript. All authors read and approved the final manuscript.

## Pre-publication history

The pre-publication history for this paper can be accessed here:


